# Supplementary material for: Pyruvate Dehydrogenase Kinase 1 inhibition mediated oxidative phosphorylation enhancement in cartilage promotes osteoarthritis progression
Source: BMC Musculoskelet Disord. 2023 Jul 20;24:597. doi: 10.1186/s12891-023-06585-6 (PMC10357736; doi:10.1186/s12891-023-06585-6)
Supplement: Supplementary file 1 — Additional file 1. [file 12891_2023_6585_MOESM1_ESM.zip › KEGG.Gsea.1653622432464/KEGG_ARRHYTHMOGENIC_RIGHT_VENTRICULAR_CARDIOMYOPATHY_ARVC.html]

Details for gene set KEGG\_ARRHYTHMOGENIC\_RIGHT\_VENTRICULAR\_CARDIOMYOPATHY\_ARVC[GSEA]

|  || Dataset | 55457\_collapsed\_to\_symbols.cls.cls #OA\_versus\_normal.cls.cls #OA\_versus\_normal\_repos |
| Phenotype | cls.cls#OA\_versus\_normal\_repos |
| Upregulated in class | normal |
| GeneSet | KEGG\_ARRHYTHMOGENIC\_RIGHT\_VENTRICULAR\_CARDIOMYOPATHY\_ARVC |
| Enrichment Score (ES) | -0.2485204 |
| Normalized Enrichment Score (NES) | -1.0210927 |
| Nominal p-value | 0.43092105 |
| FDR q-value | 0.62002 |
| FWER p-Value | 1.0 |
Table: GSEA Results Summary

  

Fig 1: Enrichment plot: KEGG\_ARRHYTHMOGENIC\_RIGHT\_VENTRICULAR\_CARDIOMYOPATHY\_ARVC      
 Profile of the Running ES Score & Positions of GeneSet Members on the Rank Ordered List

  

| SYMBOL | TITLE | RANK IN GENE LIST | RANK METRIC SCORE | RUNNING ES | CORE ENRICHMENT || 1 | SGCA | sarcoglycan alpha [Source:HGNC Symbol;Acc:HGNC:10805] | 196 | 0.824 | 0.0223 | No |
| 2 | SGCB | sarcoglycan beta [Source:HGNC Symbol;Acc:HGNC:10806] | 687 | 0.597 | 0.0113 | No |
| 3 | RYR2 | ryanodine receptor 2 [Source:HGNC Symbol;Acc:HGNC:10484] | 828 | 0.561 | 0.0260 | No |
| 4 | DSC2 | desmocollin 2 [Source:HGNC Symbol;Acc:HGNC:3036] | 1029 | 0.512 | 0.0337 | No |
| 5 | CACNA2D3 | calcium voltage-gated channel auxiliary subunit alpha2delta 3 [Source:HGNC Symbol;Acc:HGNC:15460] | 1228 | 0.471 | 0.0397 | No |
| 6 | SGCG | sarcoglycan gamma [Source:HGNC Symbol;Acc:HGNC:10809] | 1284 | 0.461 | 0.0564 | No |
| 7 | CACNA2D2 | calcium voltage-gated channel auxiliary subunit alpha2delta 2 [Source:HGNC Symbol;Acc:HGNC:1400] | 1431 | 0.439 | 0.0651 | No |
| 8 | SGCD | sarcoglycan delta [Source:HGNC Symbol;Acc:HGNC:10807] | 1781 | 0.387 | 0.0555 | No |
| 9 | ITGA4 | integrin subunit alpha 4 [Source:HGNC Symbol;Acc:HGNC:6140] | 1854 | 0.379 | 0.0672 | No |
| 10 | CTNNA3 | catenin alpha 3 [Source:HGNC Symbol;Acc:HGNC:2511] | 2030 | 0.358 | 0.0699 | No |
| 11 | ITGA1 | integrin subunit alpha 1 [Source:HGNC Symbol;Acc:HGNC:6134] | 2421 | 0.311 | 0.0537 | No |
| 12 | ACTN2 | actinin alpha 2 [Source:HGNC Symbol;Acc:HGNC:164] | 2532 | 0.299 | 0.0588 | No |
| 13 | CDH2 | cadherin 2 [Source:HGNC Symbol;Acc:HGNC:1759] | 2679 | 0.282 | 0.0602 | No |
| 14 | CACNA1F | calcium voltage-gated channel subunit alpha1 F [Source:HGNC Symbol;Acc:HGNC:1393] | 2726 | 0.278 | 0.0693 | No |
| 15 | ITGB5 | integrin subunit beta 5 [Source:HGNC Symbol;Acc:HGNC:6160] | 2823 | 0.267 | 0.0740 | No |
| 16 | CACNG4 | calcium voltage-gated channel auxiliary subunit gamma 4 [Source:HGNC Symbol;Acc:HGNC:1408] | 3065 | 0.244 | 0.0663 | No |
| 17 | DAG1 | dystroglycan 1 [Source:HGNC Symbol;Acc:HGNC:2666] | 3203 | 0.230 | 0.0662 | No |
| 18 | EMD | emerin [Source:HGNC Symbol;Acc:HGNC:3331] | 3404 | 0.211 | 0.0602 | No |
| 19 | DES | desmin [Source:HGNC Symbol;Acc:HGNC:2770] | 3570 | 0.197 | 0.0563 | No |
| 20 | CACNG2 | calcium voltage-gated channel auxiliary subunit gamma 2 [Source:HGNC Symbol;Acc:HGNC:1406] | 3819 | 0.174 | 0.0449 | No |
| 21 | CTNNA1 | catenin alpha 1 [Source:HGNC Symbol;Acc:HGNC:2509] | 4003 | 0.156 | 0.0378 | No |
| 22 | CACNA1S | calcium voltage-gated channel subunit alpha1 S [Source:HGNC Symbol;Acc:HGNC:1397] | 4031 | 0.153 | 0.0426 | No |
| 23 | CACNA2D1 | calcium voltage-gated channel auxiliary subunit alpha2delta 1 [Source:HGNC Symbol;Acc:HGNC:1399] | 4747 | 0.091 | -0.0089 | No |
| 24 | CACNG1 | calcium voltage-gated channel auxiliary subunit gamma 1 [Source:HGNC Symbol;Acc:HGNC:1405] | 4773 | 0.089 | -0.0068 | No |
| 25 | TCF7L2 | transcription factor 7 like 2 [Source:HGNC Symbol;Acc:HGNC:11641] | 5034 | 0.070 | -0.0239 | No |
| 26 | DMD | dystrophin [Source:HGNC Symbol;Acc:HGNC:2928] | 5605 | 0.023 | -0.0672 | No |
| 27 | CACNA1C | calcium voltage-gated channel subunit alpha1 C [Source:HGNC Symbol;Acc:HGNC:1390] | 6367 | -0.033 | -0.1250 | No |
| 28 | CACNB3 | calcium voltage-gated channel auxiliary subunit beta 3 [Source:HGNC Symbol;Acc:HGNC:1403] | 6753 | -0.059 | -0.1523 | No |
| 29 | DSP | desmoplakin [Source:HGNC Symbol;Acc:HGNC:3052] | 6931 | -0.074 | -0.1627 | No |
| 30 | ITGAV | integrin subunit alpha V [Source:HGNC Symbol;Acc:HGNC:6150] | 7067 | -0.085 | -0.1694 | No |
| 31 | TCF7L1 | transcription factor 7 like 1 [Source:HGNC Symbol;Acc:HGNC:11640] | 7383 | -0.109 | -0.1889 | No |
| 32 | LAMA2 | laminin subunit alpha 2 [Source:HGNC Symbol;Acc:HGNC:6482] | 7392 | -0.110 | -0.1846 | No |
| 33 | LEF1 | lymphoid enhancer binding factor 1 [Source:HGNC Symbol;Acc:HGNC:6551] | 7467 | -0.116 | -0.1851 | No |
| 34 | ACTN3 | actinin alpha 3 [Source:HGNC Symbol;Acc:HGNC:165] | 7557 | -0.124 | -0.1863 | No |
| 35 | CACNB1 | calcium voltage-gated channel auxiliary subunit beta 1 [Source:HGNC Symbol;Acc:HGNC:1401] | 7589 | -0.126 | -0.1830 | No |
| 36 | PKP2 | plakophilin 2 [Source:HGNC Symbol;Acc:HGNC:9024] | 7731 | -0.136 | -0.1878 | No |
| 37 | ITGA9 | integrin subunit alpha 9 [Source:HGNC Symbol;Acc:HGNC:6145] | 7785 | -0.140 | -0.1855 | No |
| 38 | ITGA2 | integrin subunit alpha 2 [Source:HGNC Symbol;Acc:HGNC:6137] | 7857 | -0.145 | -0.1844 | No |
| 39 | ITGB7 | integrin subunit beta 7 [Source:HGNC Symbol;Acc:HGNC:6162] | 7873 | -0.146 | -0.1789 | No |
| 40 | ACTN1 | actinin alpha 1 [Source:HGNC Symbol;Acc:HGNC:163] | 8385 | -0.184 | -0.2104 | No |
| 41 | CACNG3 | calcium voltage-gated channel auxiliary subunit gamma 3 [Source:HGNC Symbol;Acc:HGNC:1407] | 8677 | -0.207 | -0.2236 | No |
| 42 | DSG2 | desmoglein 2 [Source:HGNC Symbol;Acc:HGNC:3049] | 8998 | -0.231 | -0.2380 | Yes |
| 43 | ATP2A2 | ATPase sarcoplasmic/endoplasmic reticulum Ca2+ transporting 2 [Source:HGNC Symbol;Acc:HGNC:812] | 9015 | -0.233 | -0.2286 | Yes |
| 44 | ITGA6 | integrin subunit alpha 6 [Source:HGNC Symbol;Acc:HGNC:6142] | 9112 | -0.240 | -0.2251 | Yes |
| 45 | ITGA10 | integrin subunit alpha 10 [Source:HGNC Symbol;Acc:HGNC:6135] | 9296 | -0.256 | -0.2277 | Yes |
| 46 | GJA1 | gap junction protein alpha 1 [Source:HGNC Symbol;Acc:HGNC:4274] | 9325 | -0.258 | -0.2182 | Yes |
| 47 | ITGB8 | integrin subunit beta 8 [Source:HGNC Symbol;Acc:HGNC:6163] | 9442 | -0.268 | -0.2150 | Yes |
| 48 | ITGB6 | integrin subunit beta 6 [Source:HGNC Symbol;Acc:HGNC:6161] | 9614 | -0.284 | -0.2154 | Yes |
| 49 | ITGA7 | integrin subunit alpha 7 [Source:HGNC Symbol;Acc:HGNC:6143] | 9724 | -0.294 | -0.2105 | Yes |
| 50 | CACNA1D | calcium voltage-gated channel subunit alpha1 D [Source:HGNC Symbol;Acc:HGNC:1391] | 9867 | -0.308 | -0.2075 | Yes |
| 51 | CACNB2 | calcium voltage-gated channel auxiliary subunit beta 2 [Source:HGNC Symbol;Acc:HGNC:1402] | 9967 | -0.318 | -0.2007 | Yes |
| 52 | CACNB4 | calcium voltage-gated channel auxiliary subunit beta 4 [Source:HGNC Symbol;Acc:HGNC:1404] | 9992 | -0.320 | -0.1880 | Yes |
| 53 | JUP | junction plakoglobin [Source:HGNC Symbol;Acc:HGNC:6207] | 10101 | -0.331 | -0.1813 | Yes |
| 54 | ITGB4 | integrin subunit beta 4 [Source:HGNC Symbol;Acc:HGNC:6158] | 10159 | -0.337 | -0.1704 | Yes |
| 55 | ITGB3 | integrin subunit beta 3 [Source:HGNC Symbol;Acc:HGNC:6156] | 10310 | -0.352 | -0.1661 | Yes |
| 56 | ACTN4 | actinin alpha 4 [Source:HGNC Symbol;Acc:HGNC:166] | 10472 | -0.370 | -0.1617 | Yes |
| 57 | LMNA | lamin A/C [Source:HGNC Symbol;Acc:HGNC:6636] | 10946 | -0.424 | -0.1793 | Yes |
| 58 | ITGA8 | integrin subunit alpha 8 [Source:HGNC Symbol;Acc:HGNC:6144] | 11046 | -0.437 | -0.1671 | Yes |
| 59 | CTNNA2 | catenin alpha 2 [Source:HGNC Symbol;Acc:HGNC:2510] | 11342 | -0.477 | -0.1683 | Yes |
| 60 | ITGA2B | integrin subunit alpha 2b [Source:HGNC Symbol;Acc:HGNC:6138] | 11645 | -0.524 | -0.1679 | Yes |
| 61 | SLC8A1 | solute carrier family 8 member A1 [Source:HGNC Symbol;Acc:HGNC:11068] | 12192 | -0.642 | -0.1812 | Yes |
| 62 | ITGA5 | integrin subunit alpha 5 [Source:HGNC Symbol;Acc:HGNC:6141] | 12474 | -0.737 | -0.1695 | Yes |
| 63 | ITGB1 | integrin subunit beta 1 [Source:HGNC Symbol;Acc:HGNC:6153] | 12566 | -0.780 | -0.1410 | Yes |
| 64 | ACTG1 | actin gamma 1 [Source:HGNC Symbol;Acc:HGNC:144] | 12613 | -0.806 | -0.1079 | Yes |
| 65 | TCF7 | transcription factor 7 [Source:HGNC Symbol;Acc:HGNC:11639] | 12628 | -0.813 | -0.0719 | Yes |
| 66 | ACTB | actin beta [Source:HGNC Symbol;Acc:HGNC:132] | 12757 | -0.920 | -0.0400 | Yes |
| 67 | CTNNB1 | catenin beta 1 [Source:HGNC Symbol;Acc:HGNC:2514] | 12866 | -1.119 | 0.0026 | Yes |
Table: GSEA details [plain text format]

  

Fig 2: KEGG\_ARRHYTHMOGENIC\_RIGHT\_VENTRICULAR\_CARDIOMYOPATHY\_ARVC      
 Blue-Pink O' Gram in the Space of the Analyzed GeneSet

  

Fig 3: KEGG\_ARRHYTHMOGENIC\_RIGHT\_VENTRICULAR\_CARDIOMYOPATHY\_ARVC: Random ES distribution      
 Gene set null distribution of ES for **KEGG\_ARRHYTHMOGENIC\_RIGHT\_VENTRICULAR\_CARDIOMYOPATHY\_ARVC**
